# An antisense RNA controls synthesis of an SOS-induced toxin evolved from an antitoxin

**DOI:** 10.1111/j.1365-2958.2007.05688.x

**Published:** 2007-05-01

**Authors:** Mitsuoki Kawano, L Aravind, Gisela Storz

**Affiliations:** 1 Cell Biology and Metabolism Branch, National Institute of Child Health and Human Development, National Institutes of Health Bethesda, MD 20892, USA.; 2 National Center for Biotechnology Information, National Institutes of Health Bethesda, MD 20892, USA.

## Abstract

Only few small, regulatory RNAs encoded opposite another gene have been identified in bacteria. Here, we report the characterization of a locus where a small RNA (SymR) is encoded *in cis* to an SOS-induced gene whose product shows homology to the antitoxin MazE (SymE). Synthesis of the SymE protein is tightly repressed at multiple levels by the LexA repressor, the SymR RNA and the Lon protease. SymE co-purifies with ribosomes and overproduction of the protein leads to cell growth inhibition, decreased protein synthesis and increased RNA degradation. These properties are shared with several RNA endonuclease toxins of the toxin-antitoxin modules, and we show that the SymE protein represents evolution of a toxin from the AbrB fold, whose representatives are typically antitoxins. We suggest that SymE promotion of RNA cleavage may be important for the recycling of RNAs damaged under SOS-inducing conditions.

## Introduction

In *Escherichia coli*, a combination of approaches based on sequence conservation, structural features and direct detection has led to the identification of approximately 80 small RNAs (sRNAs) that do not encode tRNAs, rRNAs or proteins but rather are thought to be regulators (Storz and Gottesman, 2006). While the functions of only a subset of these regulatory RNAs is known, most of the characterized sRNAs act to modulate mRNA stability and/or translation by base-pairing with mRNAs that are encoded at a different chromosomal position. In general, the region of complementarity between these *trans*-encoded sRNA and the mRNA target is limited, and all of these sRNAs have been found to bind to and require the RNA chaperone Hfq for function.

In contrast to the sRNAs encoded on the bacterial chromosome, most of the characterized plasmid sRNAs are encoded opposite the genes that they regulate and thus have perfect complementarity with their target mRNAs (Wagner *et al*., 2002). Many of these *cis*-encoded sRNAs modulate the synthesis of replication proteins and thus control the copy number of the plasmids. They act by blocking ribosome binding or by promoting the formation of an mRNA secondary structure that leads to transcription termination. Other *cis*-encoded plasmid sRNAs repress the synthesis of proteins that would otherwise be toxic to the cell (Gerdes *et al*., 1997). Because the protein toxin kills cells in which the plasmid is lost, these sense-antisense pairs have been termed addiction modules or post-segregational killing systems. Perhaps the best-characterized antitoxin sRNA is the Sok antisense RNA of plasmid R1 which represses translation of the *hok* mRNA. The Sok RNA is very unstable and is quickly degraded when the R1 plasmid is lost from the cell. Under these conditions, the more stable *hok* mRNA is translated, and the Hok protein kills the cells that no longer carry the plasmid.

In addition to the Hok-Sok systems for plasmid addiction, a number of plasmids also encode toxin-antitoxin modules where the antitoxin is a protein (Engelberg-Kulka and Glaser, 1999). There are multiple classes of these protein toxin-antitoxin modules which are encoded not only on plasmids but on bacterial chromosomes as well (Anantharaman and Aravind, 2003; Pandey and Gerdes, 2005). The majority of these systems share a common gene organization – usually operons of two closely linked genes where one gene encodes an antitoxin and the other a toxin. The antitoxins are typically transcription factors that repress the toxin-antitoxin operon and also bind to and block the activity of the toxins. The toxins encoded by these modules are a very diverse group of proteins that appear to kill cells by disrupting any of several different aspects of cell physiology. In the course of bacterial evolution, operons constituting these systems have undergone a wide range of ‘mixing and matching’ of toxin and antitoxin genes, while retaining the same organizational syntax (Anantharaman and Aravind, 2003).

Free-living organisms in particular encode an abundance of these protein toxin-antitoxin modules, but their physiological roles have been controversial. It has alternatively been proposed that toxins promote cell killing, cell stasis, long-term cell persistence or reuse of resources; although some of these roles may not be mutually exclusive (Buts *et al*., 2005; Gerdes *et al*., 2005; Condon, 2006). While the physiological roles of the toxin proteins remain under debate, the biochemical functions of some of the toxins are more clearly understood. Many toxins belong to the RelE, MazF/Kid, Doc and PIN domain superfamilies of predicted RNA endonucleases, and subsets of these toxins have been shown to promote mRNA cleavage either on their own or in association with ribosomes. As for the Hok-Sok example, the protein antitoxin generally is significantly less stable than the toxin such that toxin activity increases under conditions of stress when the antitoxin is degraded.

**Fig. 1:**
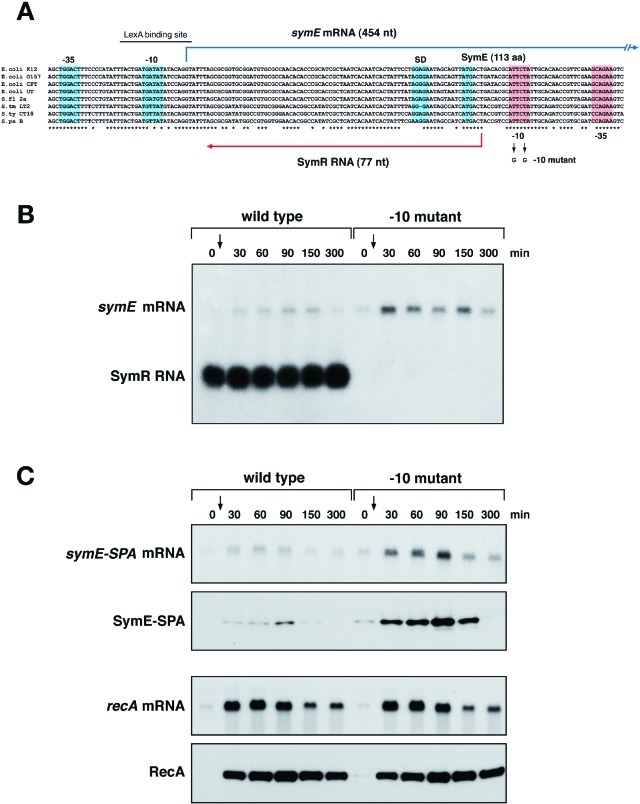
SymR RNA represses SymE translation. A. Genetic organization of the *symER* locus. Multiple sequence alignments of the different *symER* sequences were constructed using clustalw. The species correspond to *E. coli* strains K12, O157:H7, CFT073 and UT189, *Shigella flexneri* 2a, *Salmonella typhimurium* LT2, *S. typhi* CT18 and *S. paratyphi* B. The −10 and −35 promoter sequences, the Shine-Dalgarno sequence and initiation codon of *symE* are indicated by blue boxes. The −10 and −35 sequences of *symR* are indicated by red boxes. B. *symE* mRNA and SymR RNA levels in MG1655 and the −10 *symR* promoter mutant. Total RNA was isolated from wild-type MG1655 and −10 mutant strains grown in LB medium at 37°C at 0, 30, 60, 90, 150 and 300 min after treatment with 1 μg ml^−1^ mitomycin C. Samples (5 μg) were analysed by Northern hybridization using oligonucleotide probes specific to *symE* and SymR. C. *symE-SPA* mRNA and protein and *recA* mRNA and protein levels in MG1655 *symE*-SPA and the −10 *symR* promoter mutant. Total RNA and cell lysates were prepared from MG1655 *symE*-SPA and *symE*-SPA −10 mutant strains in grown in LB medium at 37°C at 0, 30, 60, 90, 150 and 300 min after treatment with 1 μg ml^−1^ mitomycin C. RNA samples (5 μg) were analysed by Northern hybridization using oligonucleotide probes specific to *symE* and *recA*, and cell lysates were analysed by immunoblot assays using monoclonal anti-FLAG M2-AP and polyclonal anti-RecA antibodies.

In our cloning-based screen for *E. coli* sRNAs, we identified a 77 nucleotide RNA, denoted RyjC based on its genomic position, which is encoded opposite the 5′ end of the *yjiW* mRNA (Kawano *et al*., 2005). The RyjC promoter is embedded in the *yjiW* coding sequence (Fig. 1A) and the sRNA and much of its sequence are conserved in *Salmonella*. Here we examine the function of the *yjiW*-RyjC sense-antisense pair which we have renamed SymE (SOS-induced *yjiW* gene with similarity to *M*az*E*) and SymR (symbiotic RNA). We present evidence that SymR serves an antitoxin function by repressing SymE synthesis and that the SymE protein has properties of the endonuclease toxins. We propose that SymE represents an instance of toxin evolution from a superfamily hitherto only known to contain antitoxin transcription factors. We also suggest that the SymE-SymR pair might represent the deployment of a chromosomal toxin-antitoxin module for the recycling of RNA damaged concomitantly with DNA.

## Results

### SymR antisense RNA represses *symE* translation

To examine the effects of SymR RNA expression on the *cis*-encoded *symE* gene, we needed to construct a strain lacking the SymR RNA. Given that the two genes overlap and the promoter of SymR is embedded within the *symE* open reading frame, we had to eliminate SymR expression by mutating the *symR* promoter. This was achieved by introducing two point mutations onto the *E. coli* chromosome that disrupted the −10 sequence of the *symR* promoter but did not alter the SymE amino acid sequence (Fig. 1A). This −10 mutation effectively abolished expression of the SymR RNA (Fig. 1B). Previous studies had shown that the *symE* (*yjiW*) promoter has a LexA binding site and is strongly induced by DNA damaging agents (Fernández De Henestrosa *et al*., 2000). We therefore compared *symE* mRNA levels in wild-type MG1655 cells and the corresponding strain carrying the −10 promoter mutation at different times before and after cells were treated with the DNA damaging agent mitomycin C (Fig. 1B). For wild-type cells virtually no *symE* mRNA expression was observed in the absence of mitomycin C treatment. Low levels of the mRNA were detected 30 min after SOS induction with a peak between 60 and 90 min. Overall slightly higher *symE* mRNA levels were detected for the −10 mutant strain; more *symE* mRNA could be detected in the absence of DNA damage, and higher induction was observed at 30, 60, 90 and 150 min. Generally, the elimination of SymR expression resulted in approximately threefold higher *symE* mRNA levels.

We also examined the effects of SymR RNA on SymE protein levels by integrating a sequential peptide affinity (SPA) tag adjacent to the SymE stop codon on the chromosomes of the wild type and −10 mutant strains. When *symE-SPA* mRNA levels after mitomycin C treatment were compared in the wild type and −10 mutant backgrounds, we again observed an approximately threefold increase in *symE-SPA* mRNA levels in the −10 mutant compared with the wild-type strain (Fig. 1C). A greater than sevenfold difference in SymE-SPA protein levels was observed between the wild-type and −10 mutant strains. For the wild-type strain, no protein was detected in the absence of mitomycin C treatment and only low levels were detected after the treatment. As for the *symE-SPA* mRNA, the peak of SymE-SPA protein was at 90 min. For the −10 mutant, SymE-SPA was clearly present in uninduced cells and the levels were strongly increased upon mitomycin C treatment. In contrast, no difference in *recA* mRNA and protein levels was observed with the same wild type and −10 promoter extracts (Fig. 1C). Together these results indicate that while the presence of the SymR RNA has some effect on *symE* mRNA levels, there is a greater effect on protein levels. The presence of the antisense RNA however, does not impact the timing of SymE expression after SOS induction.

### SymR RNA levels are 10-fold higher than *symE* mRNA levels

**Fig. 2:**
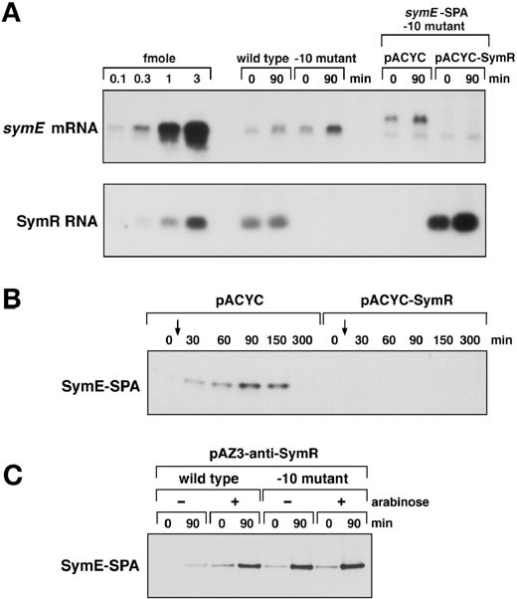
Effects of different SymR RNA levels. A. Quantitative Northern analysis of *symE* and SymR RNA levels. Total RNA (10 μg) isolated for the Northern analysis in Fig. 1B and total RNA (0.5 μg) isolated from the samples used for the Western analysis in Fig. 2B was separated alongside *in vitro* synthesized RNA (0.1, 0.3, 1 and 3 fmol) on 1.2% agarose gels. The cellular levels of *symE* mRNA and SymR RNA were determined from the ratio of the signals of control RNAs to the cellular RNAs. B. SymR RNA expressed *in trans* can repress SymE synthesis. The *symE*-SPA −10 mutant carrying pACYC and pACYC-SymR was grown to OD_600_∼0.3 in LB medium containing tetracycline at 37°C. Cell lysates prepared at 0, 30, 60, 90, 150 and 300 min after treatment with 1 μg ml^−1^ mitomycin C were analysed by immunoblot assays using monoclonal anti-FLAG M2-AP antibodies. C. Expression of an anti-antisense RNA leads to increased SymE-SPA synthesis. MG1655 P_CP18_-*araE symE*-SPA and MG1655 P_CP18_-*araE symE*-SPA −10 mutant strains carrying pAZ3-anti-SymR were grown to OD_600_∼0.2 in LB medium containing chloramphenicol at 37°C. The cultures were split and half of each culture was treated with 0.02% arabinose for 30 min (0 min). All cultures were then treated with 1 μg ml^−1^ mitomycin C for 90 min (90 min). Cell extracts prepared at the 0 and 90 min time points were analysed by immunoblot assays using monoclonal anti-FLAG M2-AP antibodies.

The relative levels of the *symE* mRNA and SymR RNA were determined by quantitative Northern analysis (Fig. 2A). The total RNA samples isolated for the 0 and 90 min time points in Fig. 1B and C were separated alongside known amounts of *in vitro* synthesized SymR and *symE* RNAs. Using this approach, the amount of *symE* mRNA in wild-type strain was determined to be 0.01 fmol μg^−1^ of total RNA at time 0 and 0.02 fmol μg^−1^ at 90 min, while the amount of SymR in wild-type strain was determined to be 0.2 fmol μg^−1^ of total RNA at both time points. Thus, the ratio of *symE* mRNA and SymR is nearly 1:10 suggesting that most of the SymR RNA in the cell is not base-paired with the *symE* mRNA. We also found the SymR RNA to be very stable; almost no decrease in SymR levels was observed even 60 min after inhibiting transcription with rifampicin treatment (data not shown).

### SymR expressed *in trans* represses SymE synthesis and anti-SymR expressed *in trans* activates SymE synthesis

To test whether SymR expressed *in trans* could also repress SymE-SPA synthesis, we moved a pACYC184 plasmid expressing *symR* from its own promoter into the −10 promoter mutant strain (Fig. 2B). In cells treated with mitomycin C, SymE-SPA was detected in the control strain carrying the vector control, similar to the wild-type strain. However, for cells harbouring pACYC-SymR, no SymE-SPA protein was detected at any time points. Thus, SymR expressed from a plasmid can also repress SymE-SPA synthesis. In the pACYC-SymR containing strains, the SymR RNA was present at 8–12 fmol μg^−1^, more than 40-fold higher than the levels expressed from the chromosome (Fig. 2A). At these high levels of SymR RNA no *symE-SPA* mRNA was detected even 90 min after mitomycin C treatment. This decrease must be resulting from effects on *symE* mRNA stability because assays of a *symE–lacZ* fusion showed that pACYC-SymR did not impact transcription from the *symE* promoter (data not shown).

We also constructed a plasmid expressing an RNA that is complementary to SymR and thus was predicted to interfere with SymR base-pairing with the *symE* mRNA. In this construct the anti-SymR RNA is under the control of the P_BAD_ promoter of pAZ3. When the anti-SymR RNA was induced by arabinose for 30 min in the wild-type background, SymE-SPA synthesis was observed even in the absence of DNA damage and was further increased by mitomycin C treatment for 90 min (Fig. 2C). These SymE-SPA levels were comparable to the levels observed in the −10 mutant in the absence of arabinose induction. SymE-SPA levels were not further elevated in the −10 mutant when anti-SymR expression was induced by arabinose. Our interpretation of these results is that anti-SymR is exerting its effects by preventing SymR base-pairing with the *symE* mRNA, and this regulation is abolished when SymR is not expressed in the −10 mutant.

### SymE protein levels are controlled by the Lon protease

**Fig. 3:**
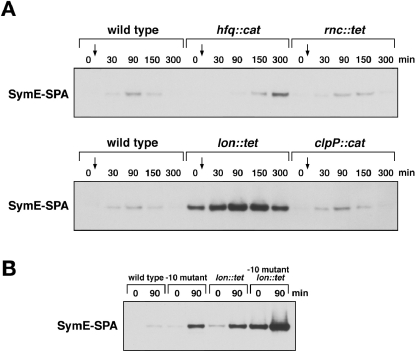
SymE protein is degraded by the Lon protease. A. SymE-SPA synthesis in *hfq*, *rnc*, *lon* and *clpP* mutant strains. The MG1655 *symE*-SPA strain and the corresponding mutant derivatives were grown to OD_600_∼0.3 in LB medium at 37°C. Cell lysates prepared at 0, 30, 90, 150 and 300 min after treatment with 1 μg ml^−1^ mitomycin C were analysed by Western hybridization using monoclonal anti-FLAG M2-AP antibodies. B. Relative SymE-SPA levels in the −10 *symR* promoter and *lon* mutant backgrounds. The MG1655 *symE*-SPA, *symE*-SPA −10 mutant, *symE*-SPA *lon* mutant, *symE*-SPA −10 *lon* mutant strains were grown to OD_600_∼0.5 in LB medium at 37°C. Cell lysates prepared at 0 and 90 min after treatment with 1 μg ml^−1^ mitomycin C were analysed by immunoblot assays using monoclonal anti-FLAG M2-AP antibodies.

For many well-characterized plasmid sRNAs that base-pair with *cis*-encoded mRNAs, the antisense RNA-target RNA duplexes are degraded by RNase III, an endoribonuclease specific for double-stranded RNA (Blomberg *et al*., 1990; Gerdes *et al*., 1992; Malmgren *et al*., 1997). In addition, all characterized *E. coli* sRNAs that base-pair with *trans*-encoded mRNA targets have been found to require the function of the Hfq RNA chaperone protein (Storz and Gottesman, 2006). To determine whether SymR regulation of SymE synthesis required either of these proteins, we moved the corresponding mutant alleles into the SymE-SPA strain and examined SymE-SPA synthesis at different time points after mitomycin C treatment (Fig. 3A). In the *rnc* mutant, the pattern of SymE-SPA synthesis was virtually identical to the wild-type strain. For the *hfq* mutant strain, we also did not detect SymE-SPA protein at 0 min. The peak of SymE-SPA synthesis was later than for the wild-type strain, but we attribute this delay to the slower growth of this mutant strain. In general, neither RNase III nor Hfq appears to be required for SymR RNA repression of SymE synthesis.

Numerous SOS-induced proteins have been found to be unusually labile to proteolysis; a feature which should allow rapid return to a non-stressed state once the DNA damage has been eliminated (Neher *et al*., 2006). Thus, we examined the effects of mutations that eliminate the expression of the ClpP and Lon proteins on SymE-SPA levels (Fig. 3A). The *clpP* mutant also was virtually identical to the wild-type strain. For the *lon* mutant strain however, the levels of the SymE-SPA protein, but not other control SPA-tagged proteins (data not shown) were significantly elevated at every time point, indicating that the SymE protein is a target of the Lon protease.

Together our results showed that synthesis of the SymE is tightly repressed at multiple levels; at the transcriptional level by the LexA repressor, at the level of mRNA stability and translation by the SymR RNA and at the level of protein stability by the Lon protease. To examine the relative contributions of each of these three regulators on SymE synthesis, we compared SymE-SPA levels in strains carrying the −10 mutant, the *lon* mutation and these two mutations in combination in the presence and absence of DNA damage (Fig. 3B). In the absence of mitomycin C treatment, SymE-SPA levels were slightly elevated in both single mutant strains, similar to levels observed in a LexA mutant strain (*lexA51*; data not shown). The SymE-SPA levels were significantly elevated in the double mutant and were induced even further upon deactivation of the LexA repressor with mitomycin C treatment indicating that the contributions of LexA, SymR and Lon to SymE repression are additive.

### SymE protein co-purifies with ribosomal proteins

**Fig. 4:**
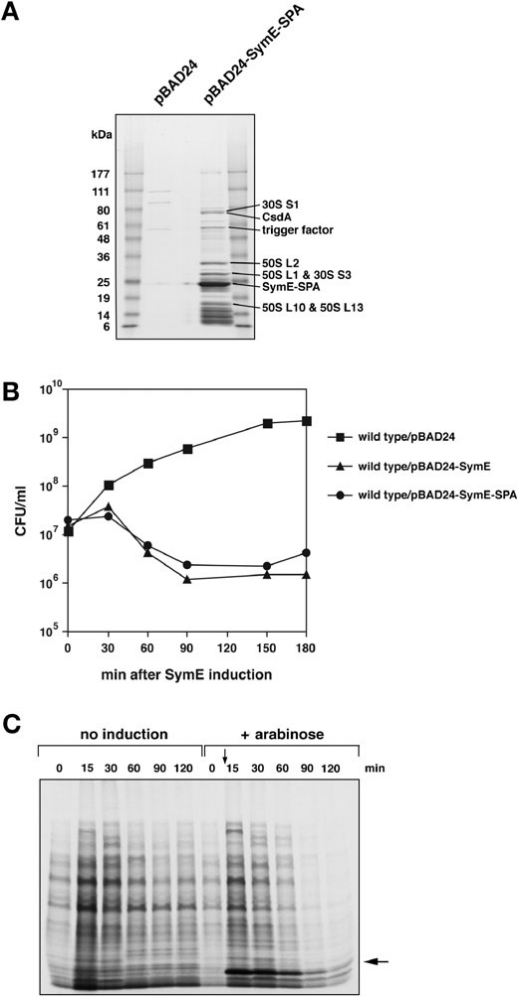
Overexpression of ribosome-associated SymE leads to reduced colony formation and decreased protein synthesis. A. Purification of SymE-SPA. Extracts prepared from MG1655 *kan*-P_CP18_-*araE* carrying either pBAD24 or pBAD-SymE-SPA were subjected to affinity purification as described in *Experimental procedures*. The samples then were analysed by 4–20% SDS-PAGE and visualized using GelCode Blue Stain Reagent. The identities of the indicated protein bands were determined by LC/MS/MS mass spectrometry. B. SymE overexpression results in reduced colony-forming ability. MG1655 P_CP18_-*araE* carrying pBAD24, pBAD-SymE and pBAD-SymE-SPA were grown to OD_600_∼0.3 at 37°C in LB medium containing ampicillin. Two min after time 0, 0.02% arabinose was added to induce transcription of *symE* or *symE-SPA*. At the indicated time points, cells were diluted and plated on LB solid medium containing ampicillin. C. SymE overexpression results in reduced protein synthesis. SDS-PAGE analysis of *in vivo* total protein synthesis after the induction of SymE. GSO120/pBAD-SymE cells were grown in M9 medium with glycerol, casamino acids and ampicillin with or without arabinose. Aliquots (0.5 ml) of the cell culture were taken at the indicated time points and added to 20 μCi of Tran^35^S-label containing ^35^S-methionine and ^35^S-cysteine as described in *Experimental procedures*. The band indicated with an arrow is SymE (∼12 kDa). This experiment was performed multiple times. The figure is a representative experiment.

To learn about the function of SymE, we purified plasmid-expressed, SPA-tagged and His-tagged SymE protein and identified co-purifying proteins (Fig. 4A and data not shown). A number of protein bands are observed for the SymE-SPA sample that are not in the vector control sample. Several of the identified bands correspond to ribosomal subunits (30S subunits S1, S3 and 50S subunits L1, L2, L10 and L13). Two other identified proteins (CsdA and trigger factor) are known to be associated with ribosomes. We also see an abundance of lower bands, which are likely to be small ribosomal proteins.

### SymE protein overexpression affects the colony-forming ability and protein synthesis

We did not observe any growth defects associated with the induced levels of SymE in either the −10 mutant strain or the strain carrying pACYC-SymR (data not shown). However, growth was markedly affected when SymE was overexpressed from the arabinose-inducible P_BAD_ promoter on a multicopy plasmid (Fig. 4B). While the number of colony-forming cells steadily increased for the vector control strain, there was a marked decrease for cells overexpressing SymE. The effects of overexpressing SymE-SPA were slightly less, but the tagged protein still clearly affected growth indicating that the SPA-tag does not dramatically alter the activity of the protein.

To further characterize the detrimental effects of SymE overexpression, we examined protein synthesis at different times after SymE induction from the P_BAD_ promoter plasmid (Fig. 4C). For each time point cells were labelled with ^35^S-methionine and ^35^S-cysteine for 1 min. Without SymE induction protein synthesis, as indicated by label incorporation, was detected at all time points. This was not the case upon SymE overproduction, where almost no protein labelling was observed at 90 and 120 min after arabinose induction. Both of these SymE overproduction phenotypes, the reduction in colony-forming ability and protein synthesis was reminiscent of phenotypes observed upon overexpression of the RelE and MazF-like toxins (Christensen and Gerdes, 2003; Christensen *et al*., 2003; Zhang *et al*., 2003). Like SymE, the RelE protein also has been shown to be associated with ribosomes (Galvani *et al*., 2001).

### SymE overexpression leads to RNA cleavage

**Fig. 5:**
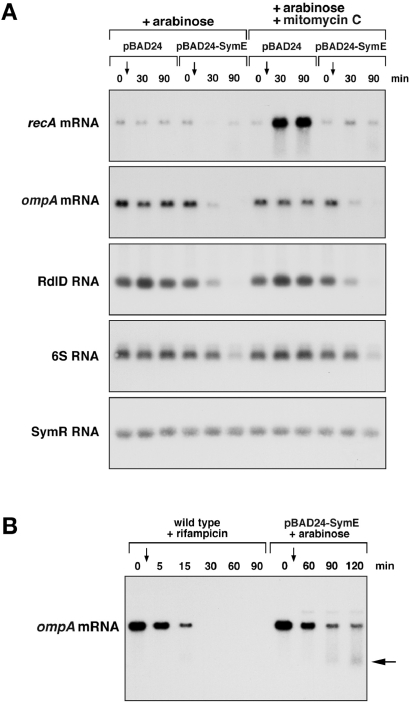
SymE exhibits ribonuclease activity. A. SymE overexpression leads to reduced levels of some RNAs. MG1655 P_CP18_-*araE* carrying either pBAD24 or pBAD-SymE were grown to OD_600_∼0.5 at 37°C in M9 minimal medium supplemented with glycerol and casamino acids. Total RNA isolated at 0, 30, and 90 min after treatment with 0.02% arabinose and 0.02% arabinose plus 1 μg ml^−1^ mitomycin C. Samples (5 μg) were analysed by Northern hybridization using oligonucleotide probes specific to *recA*, *ompA*, RdlD, 6S and SymR. B. Detection of degradation products upon SymE overexpression. MG1655 cells were grown to OD_600_∼0.5 at 37°C in LB medium, and total RNA was isolated at 0, 5, 15, 30, 60 and 90 min after treatment with 300 μg ml^−1^ rifampicin. MG1655 P_CP18_-*araE* cells carrying pBAD-SymE were grown to OD_600_∼0.25 at 37°C in LB medium, and total RNA was isolated at 0, 60, 90, and 120 min after treatment with 0.02% arabinose. Samples (5 μg) were analysed by Northern hybridization using an oligonucleotide probe specific to *ompA*. Degradation products are indicated by the larger arrow.

Overproduction of RelE and MazF as well as other related toxins leads to the degradation of mRNA molecules (Christensen and Gerdes, 2003; Zhang *et al*., 2003; 2005). To test whether the same was observed upon SymE overexpression, total RNA was isolated at different times after arabinose induction with and without mitomycin C treatment, and the levels of different mRNAs and sRNAs was examined by Northern analysis (Fig. 5A). The levels of the *recA* and *ompA* mRNAs were dramatically decreased upon SymE overexpression. The levels of two sRNAs, RdlD and 6S, also were affected, particularly at 90 min. In contrast the levels of the SymR RNA itself were not changed. For all of the RNAs, similar effects were observed with and without mitomycin C treatment. We suggest that the decrease in the *recA*, *ompA*, RdlD and 6S RNA levels most likely is resulting from SymE-promoted cleavage of these RNAs rather than general inhibition of transcription because distinct shorter *ompA* mRNA fragments were observed at 90 and 120 min after SymE induction which were not detected when transcription was inhibited with rifampicin (Fig. 5B).

The fact that RdlD and 6S RNA levels are decreased upon SymE overproduction suggests that the RNAs do not need to be actively translated in order to be targets of SymE-mediated degradation. This is supported by the observation that a *lacI* mRNA with and without a start codon mutation appear to be degraded at the same rate (Fig. S1) and is similar to what is found for MazF, which can cleave mRNAs independent of the ribosome (Zhang *et al*., 2003).

### SymE protein belongs to the AbrB superfamily

Given the functional analogies between the SymE-SymR and the toxin-antitoxin modules, we sought to understand better the evolutionary relationships between SymE and previously known protein components of toxin-antitoxin modules. A psi-blast search initiated with SymE recovers statistically significant hits (*e*-value = 10^−3^−10^−26^) to over 60 proteins from a wide range of species of different γ- and β-proteobacterial genera. In all of these proteins, the region of similarity spanned a globular domain of approximately 60 amino acids. In psi-blast searches with these globular regions, there were hits, albeit with border-line statistical significance (*e*-value ∼0.05–0.1) to proteins of the AbrB superfamily such as MazE, which function as transcription factors and antitoxins in various toxin-antitoxin modules. We also found that sensitive sequence profiles (PSSMs) and hidden Markov models (HMMs) of the AbrB domain superfamily recovered highly significant hits to SymE and its homologues (*e*-value = 10^−6^−10^−10^). In addition, sequence-structure threading of SymE using the 3PSSM program recovered the structure of the antitoxin MazE (1UB4; 1MVF).

**Fig. 6:**
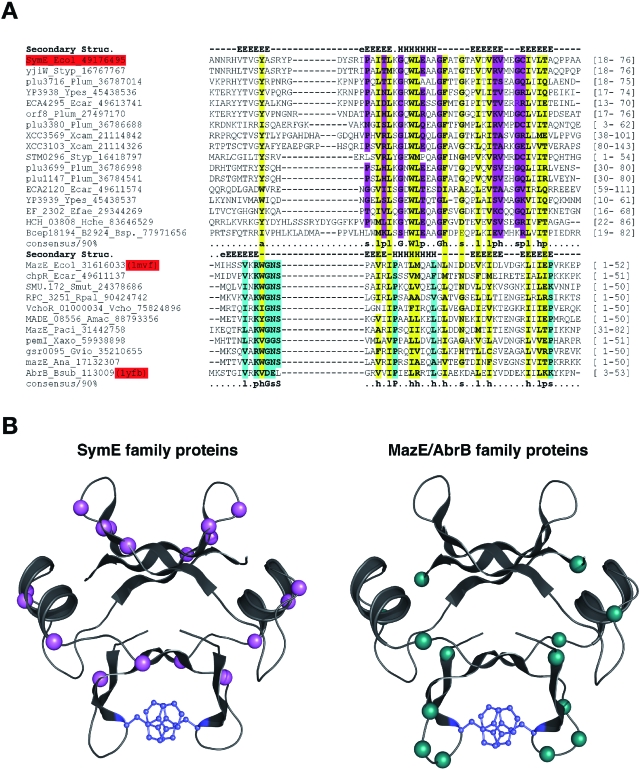
SymE is an AbrB superfamily member that has acquired a toxin-like function. A. Multiple alignment of the SymE family and other members of the AbrB superfamily. Proteins are denoted by gene name, species abbreviation, and GenBank Identifier (gi) number; separated by underscores. Positions strongly conserved at or above the 90% applied on the entire family of proteins only among the classic SymE family proteins are shaded pink, whereas those similarly conserved only in the classic MazE/AbrB family are shaded aqua and those conserved between both are shaded yellow. Consensus similarity designations are as follows: h, hydrophobic residues (ACFILMVWY); s, small residues (AGSVCDN); p, polar residues. Secondary structure assignments obtained from the jpred prediction for the SymE family and from the crystal structures (like PDB: 1mvf and 1ub4) for the rest of the AbrB superfamily are shown above the alignment where E represents a strand and H represents a helix. The region shown in the alignment spans the entire length of the DNA binding domain of the classical AbrB proteins. The boundaries are shown to the right. Species abbreviations are as given for the full alignment in Fig. S2. B. Models of the classic SymE family and classic MazE/AbrB family proteins. An idealized version of the AbrB fold was constructed using the consensus sequence derived from the hidden Markov model for the entire fold using SWISSMODEL server of SWISSPDB and the 1mvf structure as a template; in both cases the structure is depicted as a dimer formed by two interlocking monomers, which was achieved using the oligomer mode in SWISSMODEL (Guex and Peitsch, 1997). The positions that are conserved in the SymE family at the 90% consensus are coloured pink as in Fig. 6A. The majority of them line a groove on one face of the protein. This surface faces away from the surface that contains the most conserved positions unique to the classical members of the MazE/AbrB superfamily (coloured aqua). The uniquely conserved regions include polar residues that could potentially mediate the key interactions of SymE with the ribosome or target RNA. The aromatic residues involved in stabilizing the dimer through a π–π stacking interaction are shown in blue.

We prepared a multiple sequence alignment of the conserved globular domain of SymE and all its homologues (denoted SymE family) (Fig. 6A) and used the jpred program, which combines the information from a PSSM, HMM and amino-acid frequencies in the alignment to predict the secondary structure of SymE (Cuff and Barton, 2000). The predicted structure was entirely congruent to that of the AbrB fold (Fig. 6B). Comparison of multiple alignments of the SymE family with classical members of the AbrB superfamily indicated that they shared all the key hydrophobic residues, which constitute the core of this folding. These include a highly conserved hydrophobic residue in the middle of the central α-helix, and an aromatic residue in β strand-1 that forms a π–π stacking interaction critical for the characteristic dimerization of these domains. Taken together these observations strongly indicate that SymE and its homologues contain an AbrB fold and constitute a distinct family within this superfamily.

This relationship between SymE and the AbrB family was astonishing, because all previously characterized AbrB members of the toxin-antitoxin modules were transcription factors that act as antitoxins. In contrast, SymE exhibited properties that were hitherto only exhibited by toxins. A careful examination of the sequence conservation patterns and their distribution on the three-dimensional structure suggested that beyond the residues those stabilize the structural core the AbrB fold, the SymE family had acquired a novel constellation of residues (Fig. 6). These were predicted to map to regions involved in potential nucleic acid interactions and included some polar residues that might have a role in potential cleavage of RNAs. It was conceivable that SymE might antagonize the functions of other AbrB family members thereby releasing another toxin, but we observed the same decreases in colony-forming ability upon SymE overproduction in a wild-type strain and a strain lacking the five previously described toxin-antitoxin modules (data not shown). Thus, we suggest that the SymE proteins have undergone a functional shift from a transcription factor or antitoxin to an RNA-associating protein with toxin-like properties.

### Gene neighbourhood analysis of the SymE systems supports a toxin-like function

**Fig. 7:**
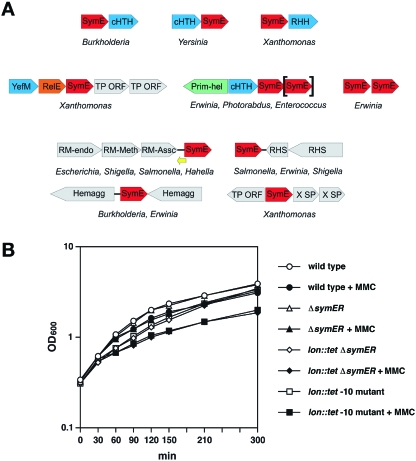
Physiological role of SymE. A. Gene neighbourhoods and predicted operons of the SymE family of genes. The orientation of the genes is denoted by the direction of the arrow. The yellow arrow denotes the SymR RNA that is transcribed in the SymE proper group. The genes are all labelled as per the gene products: SymE = SymE family; cHTH = a Cro/cI type transcriptional regulator, RHH = a MetJ/Arc-type transcription factor, YefM = a YefM-type transcription factor, RelE = a toxin homologous to the RelE toxins, Prim-Hel = the gene encoding a two module protein with a N-terminal DNAG-type primase domain and C-terminal MCM-like AAA + helicase domain. Shown in grey are other genes belonging to the larger genomic context, in the mobile islands in which the SymE family genes are inserted. These include transposons = TP ORF and restriction-modification operons = RM-endo, RM-Meth and RM-Assc for the endonuclease, methylase and accessory subunits respectively. Representative examples of neighbourhoods with the *Xanthomonas*-specific secreted protein (X SP) and the haemagglutinin and RHS cell-surface complex genes are also shown. The SymE enclosed in ‘[]’indicates the presence of an optional second SymE gene in some of these neighbourhoods. B. Δ*symER* growth after DNA damage. Wild type, Δ*symER*, *lon::tet*Δ*symER* and *lon::tet*−10 mutant cells were grown to OD_600_∼0.3 at 37°C in LB medium. Expression of SymE was induced by the addition of 1 μg ml^−1^ mitomycin C, and cell growth was monitored by measuring OD_600_ at the indicated time points.

To further investigate if SymE family proteins might have indeed acquired toxin-like function, we performed a detailed analysis of the gene neighbourhoods of SymE family members (Fig. 7A). The results of this analysis showed that SymE-family genes are often found in predicted operons together with genes encoding a transcription factor. The most commonly occurring transcription factor is a Cro/cI helix–turn–helix type protein (cHTH), which is found as the repressor and antitoxin in various toxin-antitoxin modules containing toxins of the PIN, RelE and Doc superfamilies. More infrequently, SymE family genes are found in operons encoding transcription factors of the YefM or MetJ/Arc ribbon–helix–helix type (RHH) superfamilies, which are also represented in toxin-antitoxin operons. Thus, where SymE genes are embedded in predicted operons with protein coding genes, they are always accompanied by another gene that encodes a transcription factor that typically functions as an antitoxin in the characterized toxin-antitoxin modules. This observation is consistent with the inference that, despite having an AbrB fold like the MazE antitoxins, the SymE family has undergone a functional shift to a toxin-like role. In these cases, SymE family genes most likely are regulated by means of transcriptional control.

A subset of the SymE genes, including SymE itself, did not contain any other transcription factor gene in their neighbourhood. In several of these cases there was noticeable nucleotide conservation upstream of the predicted start methionine, suggesting that members of this group are potentially all regulated by non-codings sRNAs as in the case of SymE. Thus, it appears likely that the SymE systems, like other toxin-antitoxin modules, had evolved through *in situ* operonic displacement of the transcription factor genes by genes for other such regulators or regulatory sRNAs that introduced a regulatory stricture an entirely different level.

### SymE role in the SOS response

Various physiological roles have been proposed for the antitoxin-toxin modules including altruistic killing (Aizenman *et al*., 1996), reversible stasis (Pedersen *et al*., 2002) and long-term persistence (Lewis, 2005) as well as quality control and the reuse of resources (Gerdes *et al*., 2005). To begin to examine the role of SymE in the SOS response, the *symER* coding region was replaced by the *kan* gene. Wild type and Δ*symER* mutant cells as well as *lon* mutant cells with and without the Δ*symER* deletion were treated with mitomycin C, and cell growth (Fig. 7B) and colony-forming units were assays at different time points (data not shown). We did not detect any differences between the Δ*symER* deletion and parent wild type or *lon* mutant cells in any of these assays suggesting that endogenous levels of SymE expressed after SOS induction do not play a role in altruistic killing, bacterial stasis or long-term persistence. We thus propose that a more likely role of SymE in the SOS response is the reuse of resources, in particular damaged RNAs.

## Discussion

Small, non-coding RNAs have been found to have a wide variety of regulatory functions in both bacteria and eukaryotes. We sought to define the role of SymR, a *cis*-encoded sRNA in *E. coli* and found that the antisense RNA tightly controls the synthesis of SymE, a SOS-induced protein that is also subject to degradation by the Lon protease. Surprisingly, despite homology to the AbrB superfamily of proteins which previously have been characterized as antitoxins, SymE has properties of the toxin-family of proteins. Similar to other toxins, SymE co-purifies with ribosomes, and overexpression of SymE leads to reduced colony formation, decreased protein synthesis as well as significant decreases in the levels of several RNAs tested. Sequence conservation and gene neighbourhood across bacterial species support the conclusion that this AbrB-family member has evolved to have a toxin-like function. These results have led us to suggest that SymE acts as an endoribonuclease, either on its own or in association with the ribosomes. Given its induction in response to DNA damage, we also propose that SymE might play a role in recycling RNAs damaged by the agents that induce the SOS response.

### Tight regulation of SymE synthesis

SymE synthesis is repressed at all levels; by the LexA repressor at the level of transcription, by the SymR RNA at the level of mRNA stability and translation, and by the Lon protease at the level of protein stability. Given the toxicity associated with SymE overexpression, we suggest that this tight, additive repression ensures endogenous SymE levels never rise to detrimental concentrations. Whether still other regulators modulate SymE synthesis remains to be seen.

We found several aspects of the SymR RNA regulation of SymE mRNA stability and translation to be surprising. First, at endogenous levels, the antisense RNA had a stronger impact on *symE* mRNA translation than on degradation. Because the SymR RNA is completely complementary to the 5′ end of the *symE* mRNA, there is extensive possibility for base-pairing and we expected this RNA-RNA duplex to be a good target for cleavage by RNase III. However, the absence of SymR only led to an approximately threefold increase in *symE* mRNA levels, and SymE synthesis was not affected in the *rnc* mutant strain. A second surprise was the high 10:1 SymR to *symE* ratio even after *symE* mRNA induction after DNA damage. All *symE* mRNA in the cell could be complexed with the SymR RNA. However, given that we do observe SymE protein synthesis, a subpopulation of the mRNA must not be involved in pairing or the pairing is sufficiently transient to allow occasional ribosome binding. This observation also raises the possibility that the SymR RNA has additional functions. Finally, the SymR RNA was unexpectedly stable. The levels or activities of most regulators are modulated. It could be that the SymR RNA is acting as a titrator rather than a switch. Alternatively there might be additional cellular factors that affect SymR RNA base-pairing with *symE*, and thus control the amount of SymR RNA that is active for regulation. The stability of SymR can probably be explained by the predicted structure of the RNA, which consists of two strong hairpins.

An intriguing difference between the SymE-SymR system and all previously characterized toxin-antitoxin modules is that in all other cases the antitoxin is rapidly degraded (for example the RelB antitoxin is a target of the Lon protease and the Sok antisense RNA is extremely unstable). In contrast, SymR antitoxin RNA is quite stable and in this case the toxin is target of Lon.

### Evolution of an AbrB-fold protein into a toxin

Different independent lines of evidence, sequence divergence, operon organization and experimental results imply that the SymE protein, while originating within the AbrB fold, has evolved into a protein with toxin-like properties. AbrB-type antitoxins are widely distributed throughout the bacterial superkingdom, but the distinctive SymE family is nearly exclusively found only in proteobacteria. This suggests that evolution of the endonuclease is a recent innovation within the AbrB superfamily. While it was previously known that different toxins or antitoxin might be replaced by functionally equivalent, but evolutionarily unrelated equivalents, this appears to be the first case where we observe a possible emergence of a protein with toxin-like properties within a superfamily that contains antitoxins. The barrel-like structure of the dimers and the DNA-binding properties of the AbrB fold may have favoured evolution of an RNA cleaving activity. The presence of predicted operons with two tandem genes of distinct SymE proteins (Fig. 7A) raises the possibility that such duplications might have allowed the original diversification of the fold.

We note that an astonishing range of structurally unrelated protein folds have been recruited for cleavage or degradation of RNAs in toxin-antitoxin modules. These include members of ancient RNase folds such as the PIN domain, which contains the same catalytic fold as the 5′-to 3′ exonucleases, and predicted toxins, like HicA, which contains an RNase H fold (Takagi *et al*., 2005; Anantharaman and Aravind, 2006). The RelE nuclease domain was originally reported to contain a unique α-β fold with no resemblance to other previously characterized nucleases (Takagi *et al*., 2005). However, careful structural comparisons show that it shares a common fold, including a core α(2)-β(4) topology, with the tRNA-specific RNase domain of the colicin D family. The MazF/KiD superfamily of toxins has been derived from the ancient SH3-like β-barrel fold, which includes several ancient non-catalytic RNA-binding domains like the ribosomal proteins L24/L21e and the SM domain (Anantharaman and Aravind, 2003). Doc toxins, in contrast appear to have a distinctive all-α helical metal-binding fold (Anantharaman and Aravind, 2003). In this context, the SymE family may represent convergent innovation of catalytic activity from a protein fold, whose previously characterized members were DNA-binding proteins. All together it appears that there has been a selection for similar RNA-cleaving/destabilizing functions on at least six independent occasions in evolution.

The phyletic patterns and genomic distributions of the SymE systems are strongly suggestive of movement between species as well as intragenomic proliferation and mobility. The presence of identical copies of the SymE systems in different genomes is especially indicative of recent duplications and transpositions of these operons. For example, the *Erwinia* genome has at least 14 copies (including two pairs of identical copies). The isolated presence of a SymE-like system in the Gram-positive bacterium *Enterococcus faecalis* suggests that the operons might also be laterally transferred between phylogenetically distant bacteria. Another notable feature is their genomic association with diverse mobile elements, such as restriction-modification systems, transposons and pathogenicity islands (see Fig. 7A for examples) (Kobayashi *et al*., 1999; Schmidt and Hensel, 2004). Widespread lateral mobility across bacterial genomes and association with other mobile elements are observed in other toxin-antitoxin modules. For example, a *relBE*-type system is associated with the restriction-modification system HsdS in the Gram positive bacterium *Streptococcus mutans* (Lemos *et al*., 2005). These observations suggest that SymE and other toxin-antitoxin modules are selfish elements that probably maintain themselves through toxin-dependent cell killing, and to tend proliferate within genomes and colocate to genomic hot spots harbouring other mobile elements. However, it is known that other mobile elements, like the restriction-modification systems and some transposons, on occasion, confer selective advantages to their hosts in the form of defences against DNA viruses or as DNA repair proteins and transcriptional regulators (Kobayashi *et al*., 1999; Aravind *et al*., 2000; 2005). Thus, it is possible that some toxin-antitoxin modules were recruited to control the cleavage and degradation of transcripts under specific conditions.

### Protection against RNA damage

Until now, RNA lesions have largely been ignored, primarily because damage to DNA was understood to have consequences for subsequent generations, while effects of RNA damage were transient and likely to only affect a limited number of RNA molecules. However, the universal importance of the tmRNA in bacteria and the presence of various mRNA decay mechanisms in eukaryotes, such as ‘no-go decay’ (NGD) for the degradation of mRNAs associated with stalled ribosome-mRNA complexes (Doma and Parker, 2006), indicate that damaged RNAs can also have serious consequences for the cell, in part by inhibiting ribosome function. Thus, it is perhaps not surprising that the SOS response to DNA damage would also lead to the induction of activities that could degrade damaged RNA.

Given the wide distribution of the toxin-antitoxin modules, including the SymE-like toxins, it is intriguing to ask whether other toxin-antitoxin modules are induced by and play roles in the DNA damage responses of other organisms. A transcript encoding the *E. coli hokE* gene, which shows homology to the plasmid-encoded *hok* genes, is induced during the SOS response, but it is not clear whether a functional toxin is made from this transcript (Fernández De Henestrosa *et al*., 2000). The synthesis of the SOS-induced TisB toxin also is regulated by an sRNA, in this case the adjacently encoded IstR RNA (Vogel *et al*., 2004). The function of this 29-amino acid toxin is not yet known. In addition, a LexA binding site was predicted to be downstream of the promoter of the *dinJ-yafQ* toxin-antitoxin operon in *E. coli* (Lewis *et al*., 1994), although there is no evidence for SOS induction of this operon (Fernández De Henestrosa *et al*., 2000). Because many other chromosomally encoded toxin-antitoxin genes are induced by stress conditions such as starvation that could lead to RNA damage and stalled ribosomes, we favour the model that RNA recycling is a general function of the ubiquitous toxin-antitoxin modules. Possibly many of the toxin-antitoxin-like systems of bacteria correspond to RNA degradation systems that are functionally analogous to the extensive RNA-based post-transcriptional regulatory mechanisms of eukaryotes.

## Experimental procedures

### Media and growth conditions

Cells were grown aerobically at 37°C in either Luria–Bertani (LB) medium or M9 minimal medium supplemented with 0.4% glycerol, 0.05% casamino acids, 1 mM MgSO_4_, 0.1 mM CaCl_2_ and 1 μg ml^−1^ thiamine. Antibiotics were used at the following concentrations when needed: ampicillin (50 μg ml^−1^), kanamycin (30 μg ml^−1^), chloramphenicol (25 μg ml^−1^), tetracycline (12.5 μg ml^−1^). Bacterial growth was monitored by measuring optical density at 600 nm (OD_600_).

### Bacterial strains and plasmids

Bacterial strains and plasmids used in this study are listed in Tables S1 and S2 respectively. The sequences of all oligonucleotides used to generate the strains and plasmids are given in Table S3. The *E. coli* strains are derivatives of wild-type strain MG1655 (F^–^ lambda^–^*ilvG*^–^*rfb*-50 *rph-1*).

#### SPA-tagged symE strain

The SPA tag which contains the 3 × FLAG and the calmodulin binding peptide (CBP) sequences separated by a TEV protease cleavage site was synthesized by the polymerase chain reaction (PCR) with plasmid pJL148 as a template (Zeghouf *et al*., 2004). The gel-purified PCR product was used to transform NM1100 (MG1655 mini-λ tet) to introduce the SPA tag at the end of *symE* using mini-λ Red recombination (Yu *et al*., 2000). The inserted *symE*-SPA-*kan* allele was moved onto MG1655 by P1 transduction. Subsequently, the *kan* cassette was excised by using the pCP20 plasmid (Cherepanov and Wackernagel, 1995) generating GSO114.

#### The −10 mutant strain

The −10 knockdown mutation of the *symR* promoter was constructed by using the mini-λ Red recombination method with strain NM1100 (Court *et al*., 2003) and single-stranded oligonucleotides with two silent mutations (CATTCT to CACTCC) in the *symE* open reading frame that abolish the *symR* promoter. The mini-λ tet prophage simultaneously was eliminated from this strain by a temperature shift from 30°C to 42°C, generating GSO115. The parent NM1100 strain for which the mini-λ tet prophage was similarly eliminated was used as the corresponding wild-type strain. To construct a −10 mutant derivative of the *symE*-SPA strain, the mini-λ tet allele of NM1100 was introduced into GSO114 by P1 transduction, and the −10 mutation was introduced as described above generating GSO116.

#### ΔsymER mutant strain

The coding region of the *symE* gene in strain DY330 was deleted and replaced by the *kan* gene, which was synthesized by PCR with the plasmid pKD4 as a template (Datsenko and Wanner, 2000; Yu *et al*., 2000), again using the mini-λ Red recombination method (Yu *et al*., 2000). The inserted *kan* allele was introduced into MG1655 by P1 transduction generating GSO117. Finally, the *kan* cassette was excised by using the pCP20 plasmid (Cherepanov and Wackernagel, 1995) generating GSO118.

#### Other mutant strains

To allow for more homogeneous expression from the P_BAD_ promoter on some of the plasmids used, the native *araE* promoter on the chromosome was replaced by constitutive promoter P_CP18_ by P1 transduction of the *kan*-P_CP18_-*araE* allele of strain BW27750 (Khlebnikov *et al*., 2001) into MG1655 generating GSO119. For some experiments the *kan* cassette in GSO119 was excised by using the pCP20 plasmid (Cherepanov and Wackernagel, 1995) generating GSO120. The *hfq-1::Ω* (Tsui *et al*., 1997), *rnc-14*::ΔTn*10* (Takiff *et al*., 1989), *lon146*::Tn*10* (Maurizi *et al*., 1985) and *clpP*::*cat* (Maurizi *et al*., 1990) alleles were introduced into GSO114, GSO115, GSO116 or GSO118 by P1 transduction to give GSO122-GSO128.

#### Plasmids

The *symR* promoter and coding sequence were amplified by PCR and cloned into the ScaI and BstZ17I sites of pACYC184 (Chang and Cohen, 1978) to generate pACYC-SymR. To generate a pBAD18-Cm (Guzman *et al*., 1995) derivative in which sRNA genes could easily be cloned behind the P_BAD_ promoter such that the sRNA would be expressed with the proper +1, the EcoRI site within *cat* gene was abolished by site-directed mutagenesis (GAATTC to GAATTT; Stratagene). A new EcoRI site was generated by site-directed mutagenesis downstream of P_BAD_ such that transcription would be initiated directly downstream of the EcoRI site generating pAZ3. The *symR* gene amplified and cloned into the new EcoRI and the HindIII sites in a reverse orientation to generate pBAD-anti-SymR. The coding sequences of *symE* and *symE-SPA* were PCR-amplified from MG1655 and GSO114 and cloned into the filled-in NcoI site of pBAD24 (Guzman *et al*., 1995) to generate pBAD-SymE and pBAD-SymE-SPA respectively.

### RNA analysis

In all cases, total RNA was isolated by acid hot-phenol extraction (Kawano *et al*., 2002).

#### Northern analysis

Total RNA in Formaldehyde Loading Dye (Ambion) containing 5 μg ml^−1^ ethidium bromide was denatured at 75°C for 5 min and separated on 1.2% agarose gel containing formaldehyde alongside radiolabelled RNA Perfect Markers (Novagen). The RNA was transferred to NYTRAN nylon transfer membranes (Schleicher and Schuell) by capillary action and subject to UV cross-linking. Membranes then were probed with ^32^P-labelled oligonucleotide probes (listed in Table S3) in ULTRAhyb-Oligo buffer (Ambion) at 45°C and washed as described in the manual for NYTRAN nylon transfer membranes.

#### Quantitative northern analysis

The quantitative northern analysis was performed essentially as described above except that different amounts of total RNA were analysed depending on the sample. The total RNA samples were run alongside *in vitro* synthesized control *symE* mRNA and SymR RNA. To synthesize the control RNA, DNA fragments for *symE* mRNA and SymR RNA were amplified by PCR using primers containing T7 promoter sequence (Table S3) and genomic DNA of MG1655 as a template. *In vitro* transcription were performed with the gel-purified PCR products and T7 polymerase, and synthesized *symE* mRNA and SymR were purified with MicroSpin G-50 spin columns (Amersham Biosciences) and quantified by measurement at A260.

### Protein analysis

#### Immunoblot assays

Total cell lysate was mixed with 2 × sample buffer (Sigma-Aldrich), heated at 95°C for 5 min, a fraction equivalent to the cells in OD_600_ = 0.05 was separated on Novex 10–20% Tris-Glycine gel (Invitrogen), and transferred to a nitrocellulose membrane (Invitrogen). The membranes were incubated with anti-FLAG M2-AP monoclonal antibody (Sigma-Aldrich) and polyclonal anti-RecA antibody (kindly provided by D. Camerini-Otero) to detect the SPA-tagged SymE and RecA proteins respectively. Signals were visualized using the Lumi-Phos WB (Pierce) for anti-FLAG M2-AP monoclonal antibody and HRP-conjugated secondary antibody (Amersham) together with the SuperSignal West Pico Chemiluminescent Substrate (Pierce) for the anti-RecA antibody.

#### Affinity purification of SPA-tagged protein

The protocol used to purify SymE-SPA was adapted from (Morita *et al*., 2005) with some modifications. GSO119/pBAD-SymE-SPA was grown to OD_600_∼0.4 at 37°C in 500 ml of LB medium containing ampicillin. Expression of SymE-SPA was induced by the addition of 0.02% arabinose and 0.5 μg ml^−1^ mitomycin C. After 2 h, cells (450 ml) were harvested and then washed with 20 ml of STE buffer (10 mM Tris-HCl [pH 8.0], 100 mM NaCl, 1 mM EDTA). The cell pellets were resuspended with 10 ml of IP buffer 2 (20 mM Tris-HCl [pH 8.0], 0.2 M KCl, 5 mM MgCl_2_, 10% glycerol, 0.1% Tween 20) containing Complete Mini protease inhibitor cocktail (Roche). The cell suspension was sonicated, and the cell debris was removed by centrifugation at 10 000 *g* for 1 h at 4°C. The crude extract was incubated with 500 μl of anti-FLAG M2-agarose beads (Sigma-Aldrich) overnight with rotation at 4°C. The mixture was then filtered using a poly prep chromatography column (Bio-Rad). The agarose beads were washed five times with 10 ml of IP buffer 2. Subsequently, bound proteins were eluted with 200 μl of IP buffer 2 containing 2 mg ml^−1^ 3 × FLAG peptide (Sigma-Aldrich). The purified proteins were analysed by SDS-PAGE using Novex 4–20% Tris-Glycine gel (Invitrogen), and the gel was stained with GelCode Blue Stain Reagent (Pierce).

#### Identification of proteins by mass spectrometry

Stained protein bands were excised from the gel and digested with 250 ng of sequencing grade trypsin (Roche Applied Science) in 25 μl of 25 mM ammonium bicarbonate overnight at 37°C. The digested peptides were eluted with mixture of acetonitrile and trifluoroacetic acid, and concentrated to approximately 10 μl by speed vac. The samples were desalted with ZipTip_c18_ pipette tips (Millipore). Protein identification was accomplished by automated LC/MS/MS analysis with searches of the *E. coli* protein database (http://trypsin.nichd.nih.gov/mshome.htm) using the software tool Mascot (Matrix Science).

#### Pulse-chase labelling

GSO120/pBAD-SymE cells were grown to OD_600_∼0.5 at 37°C in 50 ml of M9 medium with glycerol, casamino acids and ampicillin. Cells were then treated with 0.02% arabinose to induce symE transcription. Samples (0.5 ml) were taken at the indicated time points and added to 20 μCi of Tran^35^S-label containing ^35^S-methionine and ^35^S-cysteine (MP Biomedicals). After 1 min, 0.6 mg of unlabelled methionine and cysteine was added to the samples. After 10 more min at 37°C, cells were precipitated with 5% trichloroacetic acid. The precipitates were collected by centrifugation, washed with acetone, dissolved in about 80 μl of 2 × sample buffer (Sigma-Aldrich). Finally the samples were analysed by SDS-PAGE using Novex 10–20% Tris-Glycine gel (Invitrogen) followed by autoradiography.

### Protein sequence and structure analysis

The non-redundant (NR) database of protein sequences (National Center for Biotechnology Information, NIH, Bethesda) was searched using the blastp program (Altschul *et al*., 1997; Schaffer *et al*., 2001). Gene neighbourhoods were determined using a custom script that uses completely sequenced genomes or whole genome shot gun sequences to derive a table of gene neighbours centred on a query gene. Then the blastclust program is used to cluster the products in the neighbourhood and establish conserved co-occurring genes (http://www.ncbi.nlm.nih.gov/blast/docs/blastclust.html). These conserved gene neighbourhood are then sorted as per a ranking scheme based on occurrence in at least one other phylogenetically distinct lineage (‘phylum’ in NCBI Taxonomy database) and physical closeness (< 70 nucleotides) on the chromosome indicating sharing of regulatory −10 and −35 elements. Putative promoter regions were predicted if required by scanning for the consensus of the −10 and −35 elements in the predicted upstream regions.

Profile searches were conducted using the psi-blast program (Altschul *et al*., 1997; Schaffer *et al*., 2001) with either a single sequence or an alignment used as the query, with a default profile inclusion expectation (E) value threshold of 0.01 (unless specified otherwise), and was iterated until convergence. HMM searches were carried out using the hmm_search program of the HMMER package, after they were optimized with the HMM_caliberate program (Eddy, 1998). For all searches involving membrane-spanning domains we used a statistical correction for compositional bias to reduce false positives due to the general hydrophobicity of these proteins. Multiple alignments were constructed using MUSCLE program followed by manual adjustments based on psi-blast results (Edgar, 2004). Structural manipulations were carried out using the Swiss-PDB viewer program (Guex and Peitsch, 1997). Protein secondary structure was predicted using a multiple alignment as the input for the jpred program, with information extracted from a PSSM, HMM and the seed alignment itself (Cuff and Barton, 2000).

## Supplementary material

The following supplementary material is available for this article online:

Fig. S1 SymE overexpression leads to reduced levels of lacI mRNA with and without a start codon.

Fig. S2 Multiple alignment of the SymE family and other members of the AbrB superfamily.

Table S1 Strains used in study.

Table S2 Plasmids used in study.

Table S3 Oligonucleotides used in study.

This material is available as part of the online article from http://www.blackwell-synergy.com
