# Supplementary material for: An antisense RNA controls synthesis of an SOS-induced toxin evolved from an antitoxin
Source: Mol Microbiol. 2007 May 1;64(3):738–54. doi: 10.1111/j.1365-2958.2007.05688.x (PMC1891008; doi:10.1111/j.1365-2958.2007.05688.x)
Supplement: Fig. S1 — SymE overexpression leads to reduced levels of lacI mRNA with and without a start codon. [file mmi0064-0738-fs1.pdf]

Fig. S1. SymE overexpression leads to reduced levels of *lacI* mRNA with and without a start codon.

MG1655 P_{cp18}-*araE* and MG1655 P_{cp18}-*araE lacI*$_{GTA}$ (GSO129) carrying either pBAD24 or pBAD-SymE were grown to OD_{600} ~0.5 at 37˚C in M9 minimal medium supplemented with glycerol and casamino acids. Total RNA isolated at 0, 30, and 90 min after treatment with 0.02% arabinose (indicated by the arrow). Samples (5 µg) were analyzed by Northern hybridization using an oligonucleotide probe specific to *lacI*. The start codon mutation in *lacI* was generated in GSO120 using the mini-λ Red recombination method (Court et al., 2003) and a single-stranded oligonucleotide with a single mutation (ATG to GTA).

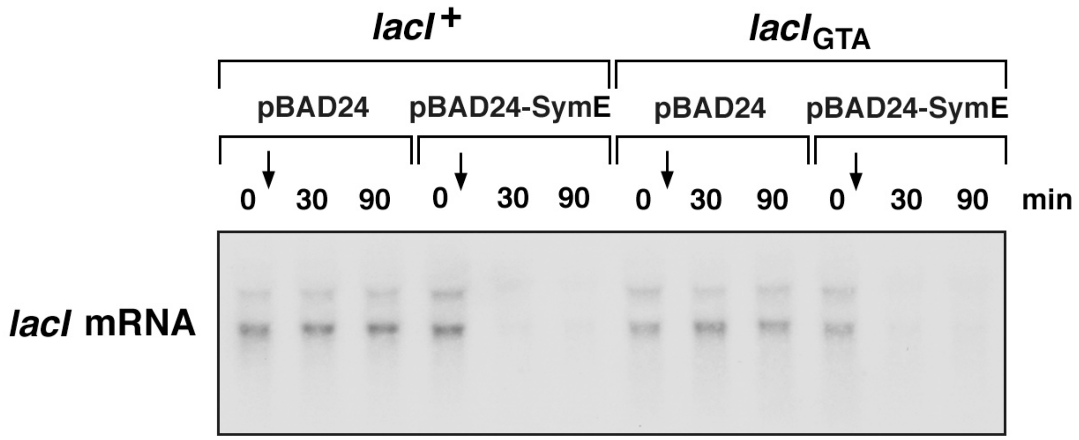

Fig. S2. Multiple alignment of the SymE family and other members of the AbrB superfamily

Proteins are denoted by gene name, species abbreviation, and gi number; separated by underscores. The gi number can be used as an unique identifier to recover the protein from the Genbank database. Amino acid residues are colored according to side chain properties and the percentage of conservation as indicated in the consensus below the alignment. Consensus similarity designations and coloring scheme are as follows: ‘h’, hydrophobic residues (ACFILMVWY) and their aromatic subset, ‘a’ are shaded yellow; ‘s’ small residues (AGSVCDN) shaded aqua and their tiny subset (GAS) are shaded green; polar residues ‘p’ are colored orange. Secondary structure assignments obtained from the JPRED prediction for the SymE family and from the crystal structures like 1mvf and 1ub4 for the rest of the AbrB superfamily are shown above the alignment where E represents a strand and H represents a helix. The boundaries of the alignment are shown to the right. Species abbreviations are as follows: *Amac : Alteromonas macleodii; Amet : Alkaliphilus metalliredigenes; Ana : Nostoc sp.; Atum : Agrobacterium tumefaciens; Bcla : Bacillus clausii; Bsp. : Burkholderia sp.; Bvie : Burkholderia vietnamiensis; Bxen : Burkholderia xenovorans; Cchl : Chlorobium chlorochromatii; Drad : Deinococcus radiodurans; Ecar : Erwinia carotovora subsp.; Ecol : Escherichia coli; Efae : Enterococcus faecalis; Gkau : Geobacillus kaustophilus; Gmet : Geobacter metallireducens; Goxy : Gluconobacter oxydans; Gura : Geobacter uraniumreducens; Gvio : Gloeobacter violaceus; Hche : Hahella chejuensis; Ipla : IncL/M plasmid; Lint : Leptospira interrogans; Maqu : Marinobacter aquaeolei; Mcel : Mycobacterium celatum; Moth : Moorella thermoacetica; Neur : Nitrosomonas europaea; Ngon : Neisseria gonorrhoeae; Nmen : Neisseria meningitidis; Nmob : Nitrococcus mobilis; Paci : Pediococcus acidilactici; Plum : Photorhabdus luminescens; Pnap : Polaromonas naphthalenivorans; Ppha : Pelodictyon phaeoclathratiforme; Ppro : Photobacterium profundum; Pput : Pseudomonas putida; Psyr : Pseudomonas syringae; Reut : Ralstonia eutropha; Rfer : Rhodoferax ferrireducens; Rnub : Roseovarius nubinhibens; Rpal : Rhodopseudomonas palustris; Sent : Salmonella enterica; Sfle : Shigella flexneri; Sfri : Shewanella frigidimarina; Smut : Streptococcus mutans; Srub : Salinibacter ruber; Styp : Salmonella typhimurium; Susi : Solibacter usitatus; Swol : Syntrophomonas wolfei; Syn : Synechococcus sp.; Vcho : Vibrio cholerae; Xaxo : Xanthomonas axonopodis; Xcam : Xanthomonas campestris; Xory : Xanthomonas oryzae; Ypes : Yersinia pestis; Ypse : Yersinia pseudotuberculosis.*

```
Secondary Struc.                 -----EEEEEE----------------eEEEEE.HHHHHHH------EEEEEE---EEEEEE-----
SymE_Ecol_49176495               ANNRHVTVGYASRYP--------DYSRIPAITLKGQWLEAAGFATGTAVDVKVMEGCIVLTAQPPAA   [ 18- 76]
SF4363_Sfle_24054893             ANNRQLTVSYASRYP--------DYSRIPAITLKGQWLEAAGFATGTAVDVKVMEGCIVLTAQPAAA   [ 18- 76]
yjiW_Sent_62182949               ANNRHLTVSYASSYP--------EYTRIPAITLKGQWLEDAGFTTGTQVDVRVMNGCIVLTAQQPQP   [ 18- 76]
yjiW_Styp_16767767               TNNRHLTVSYASRYP--------DYTRIPALTMKGQWLEAAGFATGTEVDVRVMNGCIVLTAQQPQP   [ 18- 76]
plu3716_Plum_36787014            VKPRHYTVGYRPNRG--------KPNPYPQLTIKGRWLAALGFTTGKPITITSEAGQLIIQLAEND-   [ 18- 75]
YintA_01002156_Yint_77978014     PQARKSIVGYRPNGG--------RPNPLPQLTIRGRWLEPLGFTTGQKIEVITEPGQLIIRLAVEG-   [ 13- 70]
ECA2121_Ecar_49611575            APASPVSSGYRTNGG--------KPNPLPQLTIKGRWLEALGFTKGQPVSITAEHGQLIIRTAPATK   [  7- 65]
YP3938_Ypes_45438536             KTERYYTVGYVPQRG--------KRNPPAINLKGRWLEALGFFSGQPVLITVEHGRLVIQPEIKI-   [ 17- 74]
ECA4295_Ecar_49613741            KIARYYTVGYALKNG--------KPNPPAIDLKCRWLESSGFMTGIPITVTVERRRLVIETEINL-   [ 13- 70]
ECA3402_Ecar_49612852            KTERYYTVGYASHNG--------KPNPPSAINLKGRWLEECGFITGMPVTVTVERGRIIIETQINL-   [ 17- 74]
ECA4289_Ecar_49613735            KTERYYTVGYAPHNG--------RPNPPSAINLKGRWLEESGFMTGMPITVTVERGRIVIETEINL-   [ 17- 74]
ECA3392_Ecar_49612842            QAERFTRVGYRPIK---------GNHDTPAINIAGQWLKDAGFTTGQSLKLRIMPGCIVITTQDIHE   [ 17- 74]
ECA3418_Ecar_49612868            QAERFTRVGYRPIK---------GNHATPAINIAGQWLKDAGFTTGQPLRLRIMPGCIVITTQDIHE   [ 17- 74]
orf8_Plum_27497170               KTERYYTVGYVPNGNR-------VNDATPAIHLKGQWLRQAGFNTGGQITVKVMDGCLVLIPDSEAT   [ 17- 76]
plu3380_Plum_36786688            KRDNKTIRRISQAERFGK-------VAKNGISLKGKWLQEAGFTFGMPLKIRVMPDCIVITAQNTQE   [  3- 62]
XCC3569_Xcam_21114842            RRPRQCTVSYTLYPGAHDHA---GDQHVPHVRLSGLWLQQLGFAIGTKLRITASVGRLLMEVLPPVG   [ 38-101]
XCC3103_Xcam_21114326            RTPTSCTVGYAFYEAEPGRP---HSQRIPSVRLRGLWLEQLGFAVGCKLQITARKGELVVTVERAPS   [ 80-143]
XO02821_Xory_58582444            RRPRQCTVSYTHYPGAHDHA---GDQHVPHVRLSGLWLEQLGFAIGTKLRITANAGQLLMEVLPPVE   [ 65-128]
XO01835_Xory_84367432            RTPTRCTVGYAFYDAEPGRP---DSQRIPNVRLRGLWLEQLGFAVGCKLQITARNGELVVTVAED--   [ 70-131]
XO01938_Xory_58426155            RTPTRCTVGYAFYDAEPGRP---DSQRIPNVRLRGLWLEQLGFAVGCKLQITARNGELVVTVAED--   [ 36- 97]
XO00917_Xory_58580540            RPTKQCTVGYGYYPN--------SQQRVPTLRLRGGWLEQLGFAIGSKLRVTVHDCALVIAVIDEEC   [194-252]
XO00162_Xory_58579785            RATQQCTMGYGYYPD--------SHQRVPALRLRGRWLEQLGFAIGCKLRITVRDSELVITVVGEE-   [112-169]
XO00141_Xory_84365738            RATQQCTMGYGYYPD--------SHQRVPALRLRGRWLEQLGFAIGCKLRITVRDSELVITVVGEE-   [ 63-120]
XAC1914_Xaxo_21108128            RRPRQCTVSYTHYPGAHDHA---GDQHVPHVRLSGLWLEQLGFAIGTKLRITASEGQLLMEVLPPVE   [ 65-128]
XAC2863_Xaxo_21109161            RPPQQCTIGYGYYAA--------TQQYVPTVRLRGRWLEQLGFVVGSKLDIRMREGELVMSLAHKD-   [ 60-117]
STM0296_Styp_16418797            MARLCGILTYSRV-------------ERLSVRLYGEWMPQAGFINGMPVKVRVMRDCIVITPQHTHG   [  1- 54]
STM0294.1N_Styp_39546291         NSETCMTACYSQI---------------PSQHLKGDWQEEAGFETGHGVTVKISEGCLILIAETDEV   [ 12- 63]
STY0328_Sent_16501578            MARLCGILTYSRV-------------ERLSVRLYGEWMPQAGFINGMPVKVRVMRDCIVITPQHTRG   [  1- 54]
SF0591_Sfle_24050834             DRKTGIARYYSQH---------------PSLHLKGDWLKEAGFDTGRGVTVKISEGGLTIIADSDEV   [ 30- 81]
plu3699_Plum_36786998            DRHTGMTRYYSQH---------------PSLHLKGNWLEEAGFVTGQPVQVSVEHGQLIIRLVENS-   [ 30- 80]
plu3591_Plum_36786895            DRSTGMTRYYSQH---------------PSLHLKGNWLEEAGFATGQPVQVSVEHGQLIIRLVENG-   [ 30- 80]
plu1147_Plum_36784541            DRSTGMTRYYSQH---------------PSLHLKGNWLEEAGFATGQPVQVSVEHGQLIIRLVEYN-   [ 30- 80]
ECA2120_Ecar_49611574            QQRQDLGADWVRE--------------NGGVIISGDWLTESDIARAEQLEVTAASGVIRLQRREEEV   [ 59-111]
ECA3393_Ecar_49612843            DGSETEGADWVGD--------------NGELTLAGDWLTQSGL-LGQSLSIEVLLGKITIQAERGNM   [ 61-112]
ECA3410_Ecar_49612860            DGNEAEGADWVSD--------------NGELTLAGDWLTQSGL-PDQPLIIEVLPGRITIRAVQGNM   [ 61-112]
ECA3419_Ecar_49612869            DGNEAEGADWVSD--------------NGELTLAGDWLTLSGL-LDQPLIVEVLPGKITLQAEVGAM   [ 61-112]
YPTB3619_Ypse_51591192           ENDHHIGMDWIQD--------------NGELSLSGEWLTQTGL-TGQPLAISVMAGKVIVRGQQGNM   [ 64-115]
YP3939_Ypes_45438537             LKYNNIVMAWIQD--------------NGELSLSGEWLTQTGL-TGQPLAISVMAGKVIIQFQKMNM   [ 10- 61]
EF_2302_Efae_29344269            LTVCYGHGNYKQA--------------PTQIVLQGKWLEQAGFSVGDKITVKCQQGQLTITKNETGN   [ 16- 68]
HCH_03808_Hche_83646529          QRRLKVRKGYYDYHLSSSRYDYGGFKPVPWMLIKGYWLEQAGFGVDTEVEVSVSEGRIVFTAGAG--   [ 22- 86]
ECA0399_Ecar_49609882            SQALLHLISYVSRRHADRSDMTRYYSRSPSLHLQDHWLEAAGFEIDIPVVITVEQGQLVIRPAPE--   [  9- 73]
Bcep18194_B2924_Bsp._77971656    PRTSFQTRRIVPHLKLADMA---PPVLHLWMKLSSHWIEAAGFDPEQPLKIEVMHKRLVITPIEDRR   [ 19- 82]
Bxe_A4510_Bxen_91782963          PRKPKPGRDWRTPR-------------FFSWLRIAGMWLEQAGFSPGQRVRIEVQHGRLVTTPD----   [ 23- 73]
consensus/90%                    .........a.................s.lpl.G.Wlp..Gh..s..l.lph..spl.hp......
```

```
Secondary Struc.                ..eEEEEEE-------------------EEEE-HHHHHHHH------EEEEEE---EEEEEE-----
MazE_Ecol_31616033(1mvf)        -MIHSSVKRWGNS---------------PAVRIPATLMQALNLNIDDEVKIDLVDGKLIIEPVRKEP  [  1-52]
chpR_Ecar_49611137              -MIDVPVKKWGNS---------------PAIRLSSSVMQAFDMTFNDSFDMEIRETEIALIPKKKPK  [  1-51]
SMU.172_Smut_24378686           --MQLVINKWGNS---------------SAIRLPKQLVQELQLQTNDVLDYKVSGNKIILEKVNNIP  [  1-50]
RPC_3251_Rpal_90424742          --MKVKVAKWGNS---------------LGIRLPSAAADSVGATVGSELDLTIENGELRLRSIRKTS  [  1-50]
MaquDRAFT_1702_Maqu_77953495    --MQSEIKRWGNS---------------AAVRLSSKILAQAKLDVSSPISIDVKAGKIVIEAIEKAP  [  1-50]
MazE_Ppro_11344928              -AMRTQIRKIGNS---------------LGSIIPATFIRQLELAEGAEIDVKTVDGKIVIEPIRKMK  [  1-51]
VchoR_01000034_Vcho_75824896    --MRTQIRKIGNS---------------LGNIIPATFIRQLGLVEGADIEVKTEGKRIIIEPVERQK  [  1-50]
MADE_08556_Amac_88793356        --METVIRKYGNS---------------KGVIIPAALLKELDLDVNEKIDVKTENGRIIIEPMRQLE  [  1-50]
MazE_Paci_31442758              IKEQTRLAKWGNS---------------KAARIPSQIIKQLKLDDNQDMTITIENGSIVLTPIKKNP  [ 31-82]
Gmet_2502_Gmet_78194957         --MRTNIIRIGNS---------------QGIRIPKVLLEQSHL--GAEVELEVEDEKIIIRSASRPR  [  1-48]
gsr0095_Gvio_35210655           --MKSQVARWGNS---------------LAFRIPRQIVIQLGLKPHTTLLCSVEAGALVVRPVREVK  [  1-50]
mazE_Ana_17132307               --MTTVVAKWGNS---------------LAVRIPRSIAEQAHVTEGTDINFSVEGNSIVITPKRRKK  [  1-50]
PP0770_Pput_26987506            --MQIKIQQWGNS---------------AAIRLPAAVLKQMRLGVGSTLSLDTTGETMVLKPVRSKP  [  1-50]
Psyr_0889_Psyr_63254851         --MEVKIQQWGNS---------------AAIRLPANVLKQMSLSSGDVLTLDASAEVITLKPAKAKL  [  1-50]
RPEDRAFT_3008_Rpal_77740482     --MKGQVAQWGNS---------------AALRLPKSVLEDLKVAPGSEVEMWVEAGELRVRAAPRIV  [  1-50]
NGO0517_Ngon_59800955           ---MLRLQKWGNS---------------AAVRLPARLLTALNAKIGDALETEIRNGELLIRPVKR--  [  1-47]
GK1647_Gkau_56380024            SIMTITVQKWGNS---------------LAVRIPSVIAERLALHQGSEVEMIVENQAIKLIPKKKK-  [ 10-60]
Cag_0428_Cchl_78170605          --MLTQVKKWGNS---------------FALKIPKAIVSDAKIERDSFIDINIVKGQITIENVTTQR  [  1-50]
RS9917_04555_Syn_87123100       --MKVKVQAWGNS---------------LGLRIPKVFANALGVGSGSEMEVKVEDGALLARPAAA--  [  1-48]
DR_0416_Drad_6458096            --MTSQIQKWGNS---------------LALRIPKALAQQVGLTQSSEVELLLQDGQIVIRPVPARQ  [  1-50]
PphaDRAFT_1569_Ppha_68550788    --MLTKVQKWGNS---------------LALRIPKTWALEARLENNSEVEISLVEGKLVITPVVTIK  [  1-50]
Moth_1020_Moth_83589868         IVMQPHVQKWGNS---------------LGIRIPLSLAQKIGLREGTPVDLQVDEDAIIIRRKQ---  [  3-51]
LA1780_Lint_24214480            --MESIIQKWGNS---------------LGIRIPKAMATELELNDGSHVELQYEGDKIVIYPMKK--  [  1-48]
WH5701_04345_Syn_87300647       TPSRQALRRWGNS---------------LGIRLPAAIAREARLQEDQAVELSVVEGRVLIRPVQPR-  [  6-56]
pemI_Reut_38638041              KSATLTIQKWGNS---------------LAVRIPTAVARSAHFAEGQEVEVSVDEIGVTVRPVGRRA  [  3-54]
PnapDRAFT_2207_Pnap_84713095    -MHILKLTQIGNS---------------VGVVLPKEALARLKLGKGETVFLTETPDGYTLTPYD---  [  1-48]
Rfer_3405_Rfer_89902174         -MTTLKLSQIGNS---------------VGVILPKELLARLHLEKGDVVFMTDTVNGFTISPYD---  [  1-48]
SRU_2815_Srub_83815344          MPEETTIRRIGNS---------------SGLTIPKDLLDRQHLEEGDQVHLVETEDGLLITPYD---  [  1-49]
AmetDRAFT_3460_Amet_77683837    -MKTTRLRKVGNS---------------YGFTIPKELMDKYNLNEGVELNIIEQNDGFALTPYD---  [  1-48]
ABC1016_Bcla_56909028           --MSFKVHPWGNS---------------SAVPVPSHVRKVLGIENGSEVEIEVKDGAMIVKPVKKD-  [  1-49]
XAC2188_Xaxo_21108422           --MAMQVAKWGNS---------------LAVRLPSSLVEALELREGDDIEIVVDDPRLFAVRKKPGP  [  1-50]
NE0975_Neur_30138595            --MLQTLRKAGGS---------------LVMTVPKSFIEQNGLSEGSQVELHLHGKKMIVEAPARPR  [  1-50]
pemI_Xaxo_59938898              -MHTTNLRKVGGS---------------VMLAVPPALLDVLHIGAGAKVGLAVDNGRLVVEPHARPR  [  1-51]
GuraDRAFT_3227_Gura_88935131    VEAVLGIKQWGNN---------------LGVRLPVNVARAAHLHVDQKVRIAVEEGRVIITPVDDAA  [  4-55]
MazE_Mcel_13810878              -MFTTKLRKVGGS---------------VMLAIPPALLEMLDLNAGAPVGLSVDAGQLVVDPQVRPH  [  1-51]
SF3433_Sfle_24053885            -MQTVKLRQQGGA---------------MIVTIPRDLAIDLGWSPGTELTVEKKGDSVNLRATEHKP  [  1-51]
AGR_C1711_Atum_15155939         MTVTTKIRRQGGA---------------AVMTIPPALLKMLGLEIGEQLTLEVDNGALVASPVRLEK  [ 16-67]
Moth_0253_Moth_83589122         --MDQAIIKVSSK---------------RQITIPAAIYKKLGIQPGQKLFLEVHGDKLIIWPKTKSY  [  1-50]
AbrB_Bsub_113009(1yfb)          MKSTGIVRKVDEL---------------GRVVIPIELRRTLGIAEKDALEIYVDDEKIILKKYKPN-  [  3-53]
consensus/90%                   ......l.phGsS               ..h.lP..hh..h.h..s..l.h......h.lps.....
```

Table S1. Strains used in study

Strain	Relevant genotype	Source or reference
MG1655	*E. coli* F^- λ^- *ilvG*$^-$ *rfb-50 rph-1*	lab stock
DY330	W3110 Δ*lacU169 gal490* λ*cI85* Δ(*cro-bioA*)	D. Court
NM1100	MG1655 mini-λ-*tet*	N. Majdalani
GSO114	MG1655 *symE*-SPA	this study
GSO115	MG1655 *symR-10 mutant*	this study
GSO116	MG1655 *symE*-SPA *symR-10 mutant*	this study
GSO117	MG1655 Δ*symER*::*kan*	this study
GSO118	MG1655 Δ*symER*	this study
GSO119	MG1655 *kan*-P_{CP18}-*araE*	this study
GSO120	MG1655 P_{CP18}-*araE*	this study
GSO122	MG1655 *symE*-SPA *hfq-1::*Ω	this study
GSO123	MG1655 *symE*-SPA *rnc-14*::ΔTn*10*	this study
GSO124	MG1655 *symE*-SPA *lon146*::Tn*10*	this study
GSO125	MG1655 *symE*-SPA *clpP*::*cat*	this study
GSO126	MG1655 *symE*-SPA *symR-10 mutant lon146*::Tn*10*	this study
GSO127	MG1655 Δ*symER lon146*::Tn*10*	this study
GSO128	MG1655 *symR-10 mutant lon146*::Tn*10*	this study

Table S2. Plasmids used in study

Plasmid	Resistance markers	Source or reference
pACYC184	(Tc^R)	Chang and Cohen, 1978
pACYC-SymR	(Tc^R)	this study
pAZ3	(Cm^R)	this study
pAZ3-anti-SymR	(Cm^R)	this study
pBAD24	(Ap^R)	Guzman et al., 1995
pBAD24-SymE	(Ap^R)	this study
pBAD24-SymE-SPA	(Ap^R)	this study
pJL148	(Ap^R)	Zeghouf et al., 2004
pKD4	(Km^R)	Datsenko and Wanner, 2000
pCP20	(Ap^R, Cm^R)	Cherepanov and Wackernagel, 1995

Table S3. Oligonucleotides used in study

Name	Sequence (5' to 3', with relevant restriction sites underlined)	Use
yjiW-AR20	CGCATAACCGACGGTGACATGACGG	*symE* mRNA Northern
RyjC-R	AGCGCGGTGCGGATGTGCG	SymR RNA Northern , SymR RNA synthesis
Stm-F	CAATCACTATTTAGGGAATAGCCATCATGACT	SymR RNA (S.tm) Northern
recA-R	TCGTCGATAGCCATTTTTACTCC	*recA* mRNA Northern
ompA-R	GCCACTGCAATCGCGATAGCTGTCTTTTTCAT	*ompA* mRNA Northern
RdlD-R	GCTAAGCTTAGAAAACCCCCGCACGTTGCAGGTATG	RdlD RNA Northern
6S-R	CCAACCGCGGAGCGCCACATTCTTGTGGTATG	6S RNA Northern
lacI-R	CAGCTCCGCCATCGCCGCTTCC	*lacI* mRNA Northern
TF-yjiW	GCAGGTGCAGGCGTTTATTGGAGTGATTGCCGGTAAACAGAAAGTCGCGTCCATGGAAAAGAGAAG	GSO114 PCR
TR-yjiW	CTATTGAGTCAGTGGAAGGCTCCCGGTGGTTAACCGGGAGTAAACGCTGCATATGAATATCCTCCTTAG	GSO114 PCR
yjiW-P1KO	CCCGCATCGCTAATCACAATCACTATTCCTGGAGAATAGCAGTTTAGCCATGGTCCATATGAATATCC	GSO117 PCR
yjiW-P2KO	TGGAAGGCTCCCGGTGGTTAACCGGGAGTAAACGCTGTTAGTGTAGGCTGGAGCTGCTTC	GSO117 PCR
P-10F-PAGE	CACAATCACTATTCCTGGAGAATAGCAGTTATGACTGACACGCACTCCATTGCACAACCGTTCGAAGCAGAAGTCTCCCCGGCAAATAACCGTC	GSO115 recombineering
lacI-GTA	GATAGCGCCCGGAAGAGAGTCAATTCAGGGTGGTGAATGTAAAACCAGTAACGTTATACGATGTCGCAGAGTATGCCGGTG	$lacI_{GTA}$ mutant
yjiW-Eco2	TGAGAATTCAGGGTGATGGCGGGAATACG	pACYC-SymR PCR
yjiW-F2	TATGAATTCGTATTTAGCGCGGTGCGGATGTGC	pACYC-SymR PCR
AryjC-Eco	CAGGAATTCAGCGCGGTGCGGATGTGCGCCAAC	pAZ3-anti-SymR PCR
AryjC-Hin	CAAAAGCTTAAAAAAAAGTCCTAACTGCTATTCTCCAGG	pAZ3-anti-SymR PCR
YjiW-NS	ACTGACACGCATTCTATTGCACAACC	pBAD-SymE & pBAD-SymE-SPA PCR
YjiW-C	TTACGCGACTTTCTGTTTACCG	pBAD-SymE PCR
SPA-C	CTACTTGTCATCGTCATCCTTGTAGTCG	pBAD-SymE-SPA PCR
yjiW-5-T7	TAATACGACTCACTATAGGGTATTTAGCGCGGTGCGGATGTGC	symE mRNA synthesis
YjiW-3	GAAGGCTCCCGGTGGTTAACC	symE mRNA synthesis
AyjiW-F-T7	TAATACGACTCACTATAGGGAGTCATAACTGCTATTCTCCAGG	SymR RNA synthesis
